# Supplementary material for: Fast simulation of identity-by-descent segments
Source: Bull Math Biol. 2025 May 23;87(7):84. doi: 10.1007/s11538-025-01464-8 (PMC12102126; doi:10.1007/s11538-025-01464-8)
Supplement: Supplementary file 1 — (pdf 1089 KB) [file 11538_2025_1464_MOESM1_ESM.pdf]

Supplementary figures

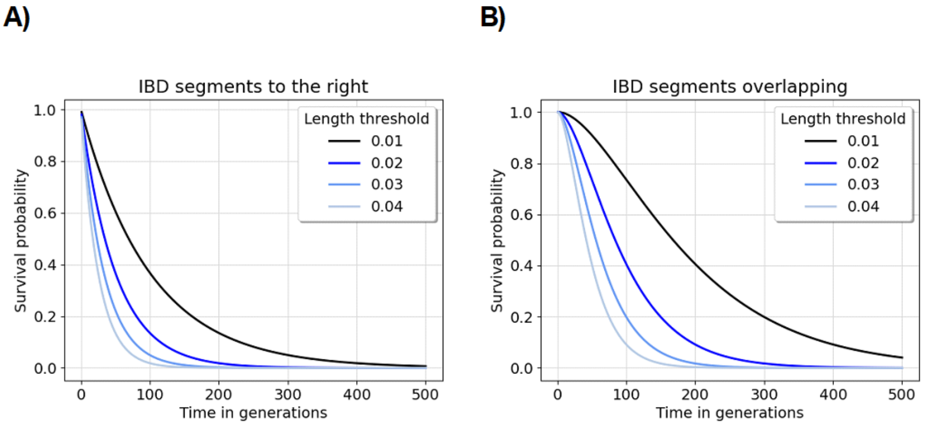

Fig. S1 The upper tail probabilities of Gamma random variables. Subplots A) and B) show the survival probabilities for shape parameters 1 and 2, respectively. The rate of the random variables is the coalescent time in generations (x-axis). The survival probability (y-axis) comes from Equations 6 and 7. The length thresholds are denoted by different colors and line styles, defined in the legend.

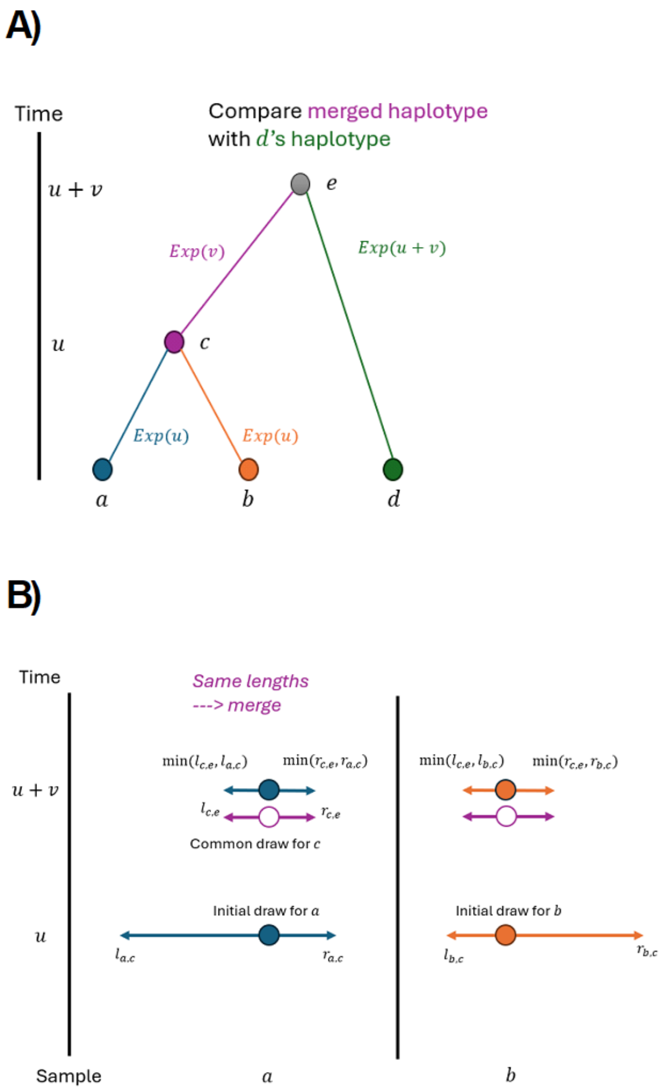

Fig. S2 Illustration of merging haplotypes. A) We draw recombination endpoints to the left and right of the focal location from Exponential(u) for both sample haplotypes a and b at coalescent time u. We draw recombination endpoints to the left and right of the focal location from Exponential(v) for the common ancestor c of a and b at coalescent time $u + v$. Colors denote branch lengths and recombination endpoints corresponding to a given haplotype. $l_{a,c}$ and $r_{a,c}$ denote the endpoints for a drawn to the left and right of the focal location at time u (lowercase denotes observation of random variables). B) We compute minimums of lengths drawn for c and a and c and b, respectively. We merge the sample haplotypes a and b in future calculations because the minimum lengths are both the recombination endpoints drawn from the common ancestor c. When comparing recombination endpoints at time $u + v$ with those of the haplotype d, we make one comparisons. The haplotypes remain longer than the detection threshold w Morgans.

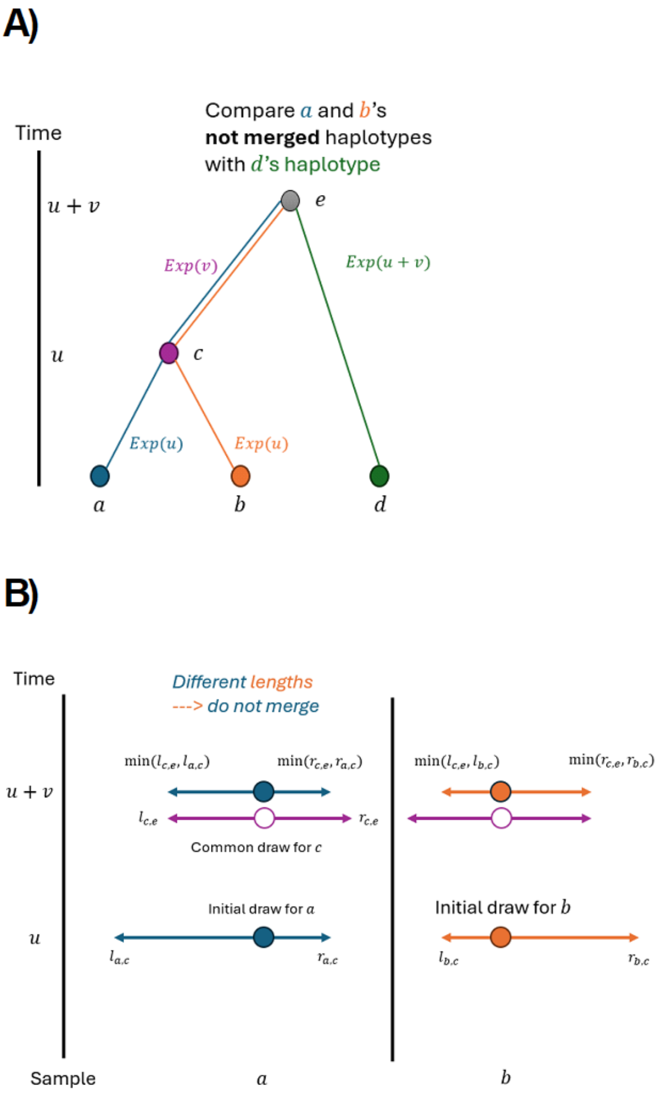

Fig. S3 Illustration of ***not*** merging haplotypes. A) We draw recombination endpoints to the left and right of the focal location from Exponential(u) for both sample haplotypes a and b at coalescent time u. We draw recombination endpoints to the left and right of the focal location from Exponential(v) for the common ancestor c of a and b at coalescent time $u + v$. Colors denote branch lengths and recombination endpoints corresponding to a given haplotype. $l_{a,c}$ and $r_{a,c}$ denote the endpoints for a drawn to the left and right of the focal location at time u (lowercase denotes observation of random variables). B) We compute minimums of lengths drawn for c and a and c and b, respectively. We ***do not*** merge the sample haplotypes a and b in future calculations because the minimum lengths are ***not*** both the recombination endpoints drawn from the common ancestor c. When comparing recombination endpoints at time $u+v$ with those of the haplotype d, we make ***two*** comparisons. The haplotypes remain longer than the detection threshold w Morgans.

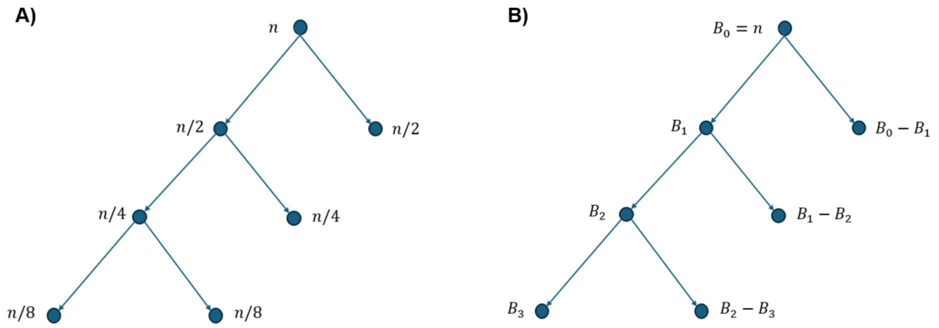

Fig. S4 Illustration of worst-case subtree sizes in random bifurcating tree. A) The worst-case subtree sizes of ancestors (dots) are when each bifurcation is an even split. B) The model $\{B_j\}$ of subtree sizes down one branching path is defined as random variables. The sample size is denoted as n.

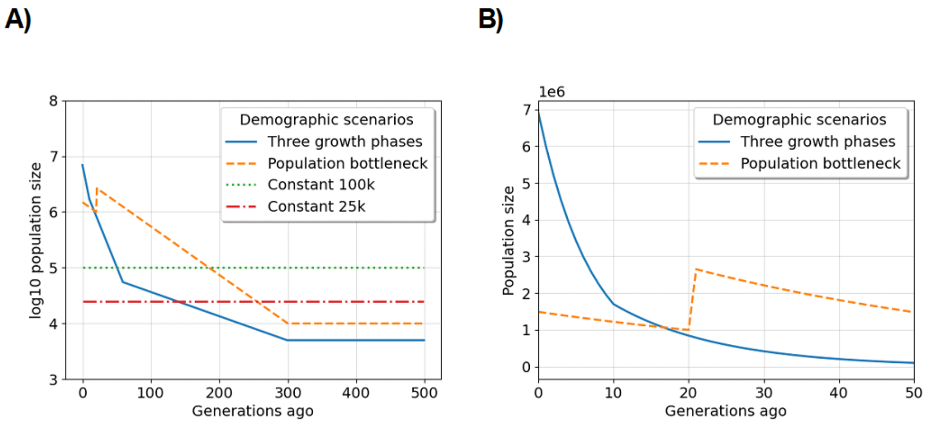

Fig. S5 Demographic scenarios we consider in simulation studies: A) coalescent time in generations ago by the log 10 population size, and B) the most recent fifty generations by population size for examples of exponential growth. The legends specify the color and line style for each scenario. As opposed to coalescent time used in the main text, we describe the scenarios moving forward in time here. The three phases of exponential growth model is as follows: a population of ancestral size 5000 diploids increases exponentially each generation at rates one, seven, and fifteen percent starting three hundred, sixty, and ten generations ago. This demographic model is similar to the "UK-like" model in Cai et al. (2023). The population bottleneck model is as follows: a population of ancestral size 10,000 diploids increases exponentially each generation at a rate of two percent starting three hundred generations ago, but twenty generations before the present day, the population experiences an instantaneous reduction in size to one million diploids. Otherwise, the demographic scenarios we explore here are populations of constant size 25,000 and 100,000 diploids.

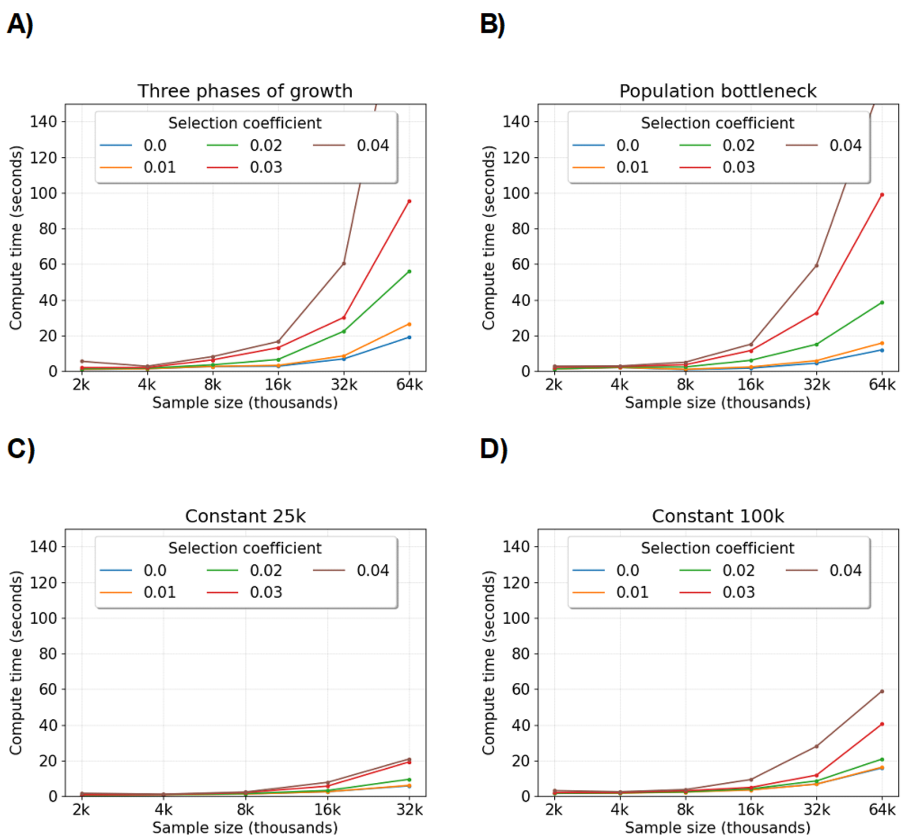

Fig. S6 Compute time to simulate IBD segment lengths around a locus depending on demography and selection. Compute time (y-axis) in seconds by sample size (x-axis) in thousands is averaged over five simulations. The legends denote colored line styles for different selection coefficients. A), B), C), and D) show results for demographic scenarios of three phases of exponential growth, a population bottleneck, and constant population sizes of 25,000 and 100,000 diploids, respectively. The Morgans length threshold is 0.01.

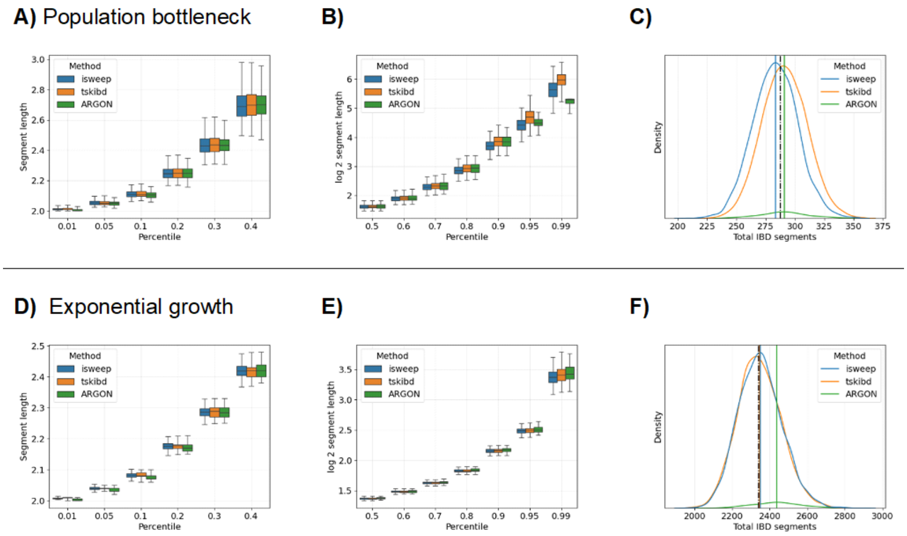

Fig. S7 Benchmarking the accuracy of segment length distributions. From simulations, we estimate the percentiles of the IBD segment length distributions in the A,B) population bottleneck and D,E) three stages of exponential growth scenarios. Box plots show the 1st, 25th, 50th, 75th, and 99th percentiles of estimates. C,F) We plot kernel densities for the total count of (detectable) IBD segments, where the solid lines are means over replicates and the black dashed line is the approximate probability of detecting an IBD segment. The legends denote results from the different simulation methods. Our method is referred to as `isweep`. Note that the y-axis scales vary to accentuate the similar results across methods. The IBD segment detection threshold is 0.02 Morgans.

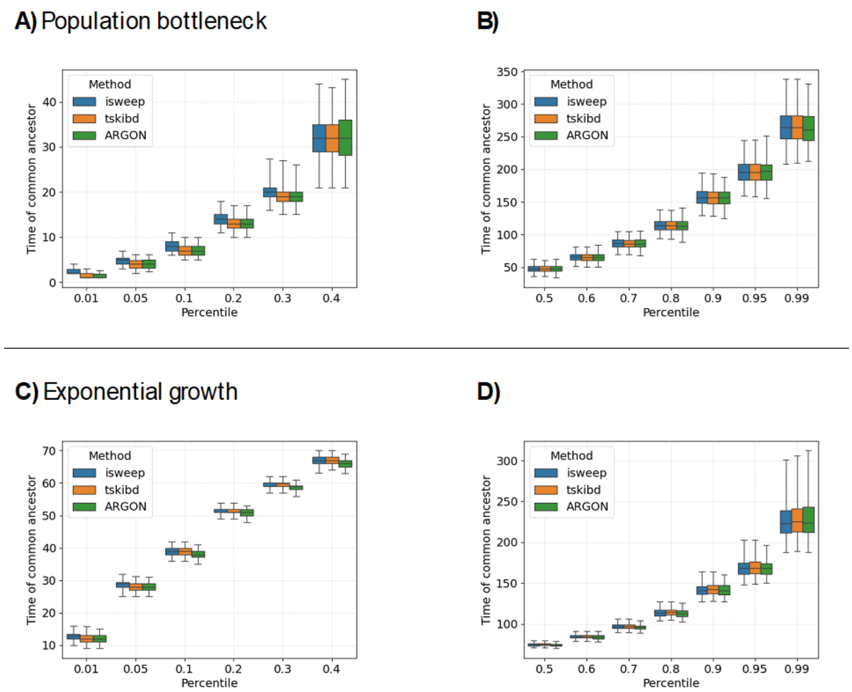

Fig. S8 Benchmarking the accuracy of time of common ancestor distributions. From simulations, we estimate the percentiles of the time of detectable IBD segment's common ancestor distributions. Box plots show the 1st, 25th, 50th, 75th, and 99th percentiles of estimates. The legends denote results from the different simulation methods. Our method is referred to as `isweep`. A,B) and C,D) show results for the population bottleneck and three stages of exponential growth scenarios. Note that the y-axis scales vary to accentuate the similar results across methods. The IBD segment detection threshold is 0.02 Morgans.

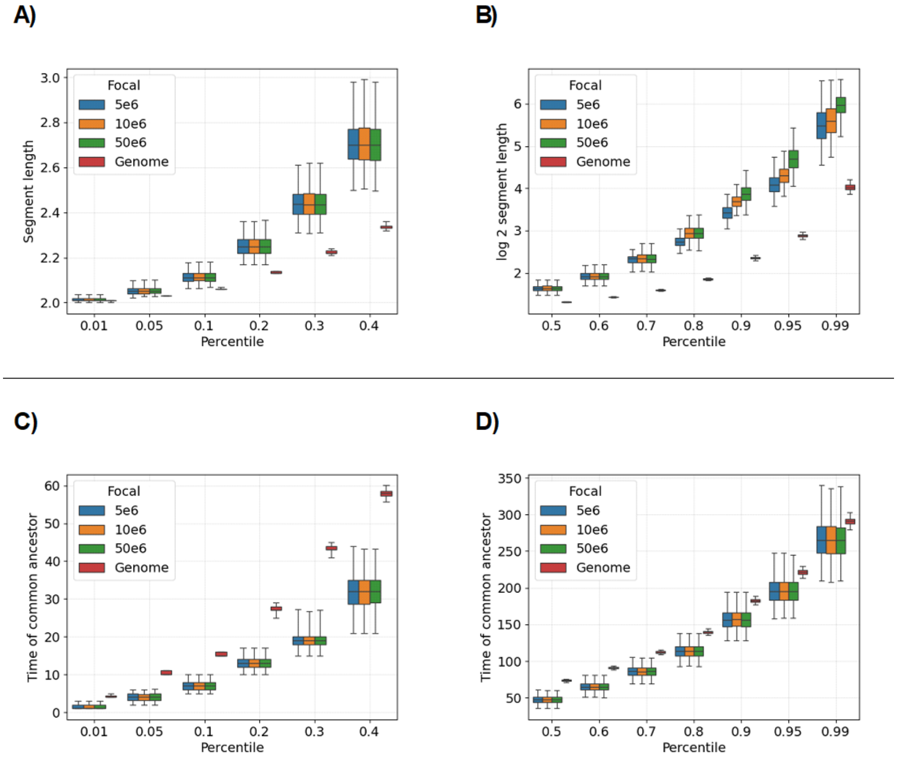

Fig. S9 IBD segments on finite chromosomes. From `tskibd` simulations, we estimate the percentiles of an IBD segment's A,B) length and C,D) common ancestor time in the population bottleneck scenario. Box plots show the 1st, 25th, 50th, 75th, and 99th percentiles of estimates. The legends denote the base pair of the focal point for 100 Mb chromosomes with a recombination rate of 1e-8. "Genome" refers to results from all IBD data along the chromosome. Note that the y-axis scales vary to accentuate the similar results across methods. The IBD segment detection threshold is 0.02 Morgans.

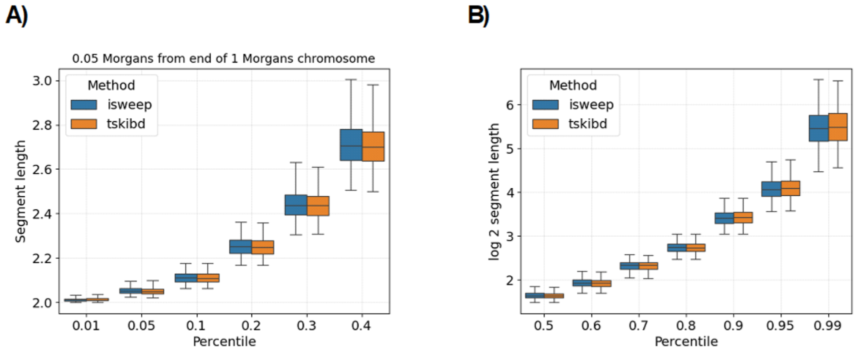

Fig. S10 Local simulation of IBD segments near chromosome ends. From `isweep` and `tskibd` simulations, we estimate the percentiles of an IBD segment's length when the focal point is 0.05 Morgans from the left end of a 1 Morgans chromosome. Box plots show the 1st, 25th, 50th, 75th, and 99th percentiles of estimates. Note that the y-axis scales vary to accentuate the similar results across methods. The IBD segment detection threshold is 0.02 Morgans.

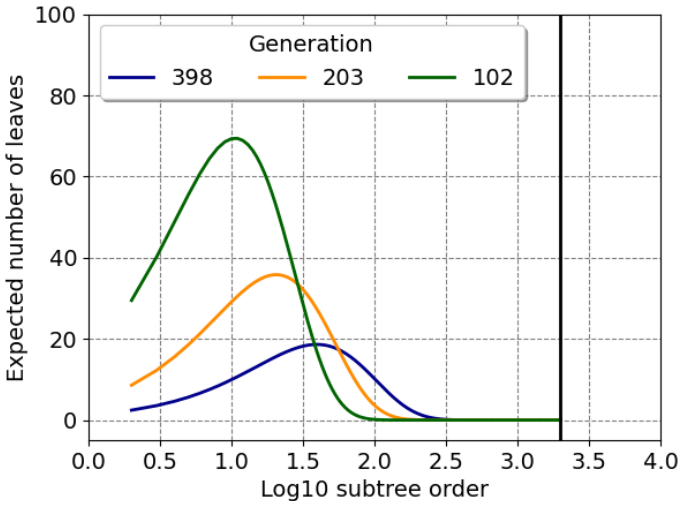

Fig. S11 The expected cardinality of subtree sizes at different coalescent times. Using Lemma 1 in Dahmer and Kersting (2015), we compute the expected number of subtrees containing r samples (x-axis) at the $(n-k)^{\text{th}}$ coalescent event. We multiply these moments by r to get the expected number of leaves under such subtrees (y-axis). There are 2000 samples. Dark blue, orange, and green lines correspond to k =50, 95, and 180. We compute the expected time of the $(n-k)^{\text{th}}$ coalescent event (Hein et al., 2005) and multiply by a population size of 10,000 to get generations (legend). The vertical line is logarithm 10 of sample size.
